# High-resolution estimates of social distancing feasibility, mapped for urban areas in sub-Saharan Africa

**DOI:** 10.1038/s41597-022-01799-0

**Published:** 2022-11-18

**Authors:** Heather R. Chamberlain, Attila N. Lazar, Andrew J. Tatem

**Affiliations:** WorldPop, School of Geography and Environmental Science, University of Southampton, Southampton, UK

**Keywords:** Geography, Developing world

## Abstract

Social distancing has been widely-implemented as a public health measure during the COVID-19 pandemic. Despite widespread application of social distancing guidance, the feasibility of people adhering to such guidance varies in different settings, influenced by population density, the built environment and a range of socio-economic factors. Social distancing constraints however have only been identified and mapped for limited areas. Here, we present an ease of social distancing index, integrating metrics on urban form and population density derived from new multi-country building footprint datasets and gridded population estimates. The index dataset provides estimates of social distancing feasibility, mapped at high-resolution for urban areas across 50 countries in sub-Saharan Africa.

## Abstract (summary)

**Table:** 

Measurement(s)	social distancing feasibility • physical distancing feasibility
Technology Type(s)	geospatial analysis
Sample Characteristic - Environment	urban areas
Sample Characteristic - Location	Sub-Saharan Africa

## Background & Summary

Since the start of the COVID-19 pandemic, public health measures intended to control disease spread have been used with near-global application. Amongst these measures, social distancing (also referred to as physical distancing) has been widely advised, with individuals required to maintain physical distance between themselves and others outside their household. Distances specified by governments have typically been between 1 and 2 metres or 6 feet^1^. Social distancing guidelines or requirements have been adopted as a public health measure or non-pharmaceutical intervention (NPI), intended to slow or stop transmission, in nearly all countries globally during the COVID-19 pandemic. Universal application of such requirements assumes that social distancing is possible everywhere, however the feasibility of populations being able to comply with social distancing requirements can vary geographically, both between and within countries^2,3^. Contextual factors affecting social distancing feasibility include population density, urban form and the built environment and a range of socioeconomic factors, such as occupation, reliance on daily wages, shared water, sanitation and hygiene facilities or dependence on public transport. In urban areas particularly, high population and built densities, can mean social distancing is all but impossible in some locations^2,4–6^.

Locations where population density and urban form are constraints for social distancing, have not been systematically identified. Existing maps or datasets related to social distancing feasibility have been limited to individual cities or single countries at most, with approaches predominantly focussing on urban form and infrastructure. For cities in a number of countries, maps considering pavement (sidewalk) width as a social distancing constraint for pedestrians have been developed, for example New York (https://www.sidewalkwidths.nyc/), London (https://www.underscorestreets.com/social-distancing) and various cities in the Netherlands (https://covid19.social-glass.tudelft.nl/). These maps have been possible due to the availability of highly-detailed data on urban infrastructure, including pavement widths. Generally such data with sufficient spatial coverage is only available for individual cities or countries with advanced geospatial data systems.

Other approaches have been used to map and identify social distancing constraints in low- and middle-income countries. Macharia *et al*.^7^ developed a sub-national social vulnerability index for COVID-19 for Kenya. Although this index doesn’t specifically consider social distancing feasibility, it includes measures related to socioeconomic deprivation, associated with difficulty in practising social distancing, such as informal employment and shared sanitation facilities, alongside the proportion of the population resident in informal settlement and internally-displaced person camps^7^. The index was applied at the sub-county level (administrative unit level 2), highlighting sub-national variations in vulnerability, but not at the intra-urban scale. Bhardwaj *et al*.^8^ used data on population density and estimates of building height, alongside access to WASH facilities to identify “hotspots for contagion and vulnerability” in Kinshasa, Cairo and Mumbai^8^. In identifying high risk locations, building height data were used to calculate an adjusted population density measure considering “livable floor space”, based on assumptions of the height of a floor. However, these adjusted population density estimates may be highly uncertain, given that the input gridded population datasets (from WorldPop or Facebook) do not consider building height in the spatial allocation of population to grid cells^9^. For informal settlements in South Africa, Gibson and Rush^10^ identified challenges in social distancing, based on the small distance between dwelling units. These studies utilised building outlines that were manually digitised from satellite imagery, and therefore were limited to a few locations due to data availability.

Given the identified data gap, this paper outlines an ease of social distancing index for sub-Saharan Africa, intended to identify locations where social distancing in urban areas is likely to be very difficult. The index incorporates residential population density and urban form metrics, which are calculated from new geospatial datasets available for sub-Saharan Africa, with index values mapped for small spatial units within urban areas. The geographic variation in social distancing feasibility highlighted by this dataset is relevant in planning disease response efforts, for both COVID-19 and future pandemic preparedness, and also can be beneficial for urban planning, development and risk identification. The datasets produced^11,12^ are available through the WorldPop Open Population Repository (https://wopr.worldpop.org/?/SocialDistancing).

## Methods

**Fig. 1:**
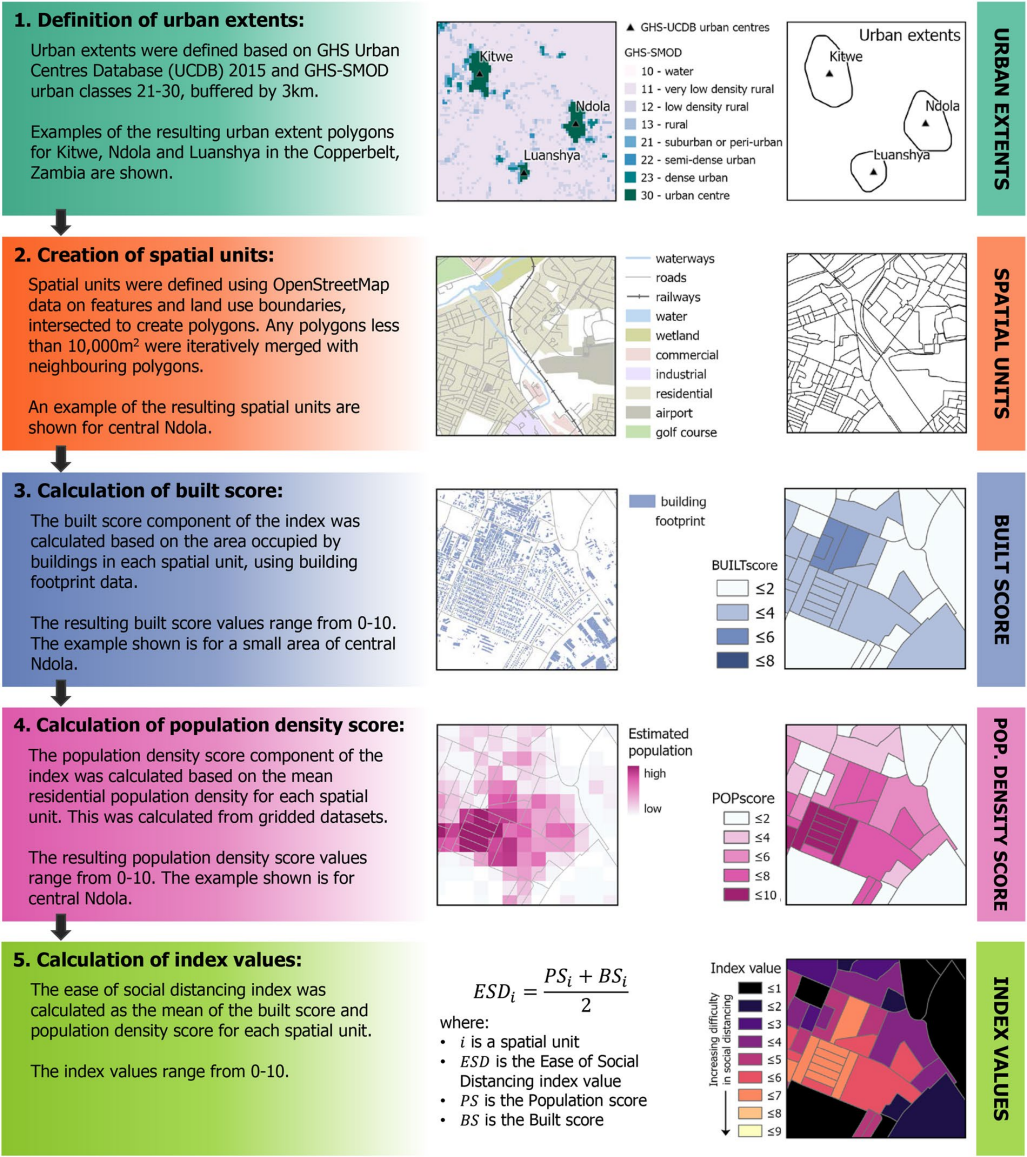
An overview of the data processing steps involved in estimating the ease of social distancing index for urban areas in sub-Saharan Africa. These steps consist of: (1) defining urban extents, (2) creating spatial units, (3) calculating the built score and (4) population density score for each spatial unit, and (5) from these scores, calculating the index values. The colours representing each step are used in the subsequent figures also.

To calculate ease of social distancing index values for small spatial units, the following steps were taken: 1) definition of urban extents; 2) creation of spatial units within urban extents; 3) calculation of the built score component of the index from building footprint data; 4) calculation of the population density score component of the index from gridded population data; and 5) calculation of the index values from the built score and population density score components for each spatial unit. A schematic overview of this process is provided in Fig. 1.

### Defining urban extents

The ease of social distancing index has been calculated for urban areas in 50 countries/territories/dependencies in sub-Saharan Africa. The index is calculated specifically for urban areas due to the high and growing proportion of the population resident in urban areas^13,14^, and there generally being greater social distancing constraints in urban settings. The urban areas for which index values have been calculated, are based on two GHSL (Global Human Settlement Layer) datasets: GHS-SMOD v2.0^15^ and GHS Urban Centre Database (UCDB) 2015 v1.2^16^. The GHS-SMOD dataset is a raster of settlement types, while the GHS-UCDB dataset provides locations (points and polygons) of urban centres. The GHS-UCDB urban centre polygons correspond to the urban centre (class 30) in the GHS-SMOD dataset. The GHS-UCDB dataset was used as the basis for selecting urban centres for inclusion in the index dataset, with the GHS-SMOD dataset used to define the extent of the urban area surrounding each urban centre.

The GHS-UCDB dataset includes a data quality field (QA2_1V); all urban centres in the GHS-UCDB dataset classified as being true positives (QA2_1V = 1) were selected for inclusion in the index dataset. For these selected urban centres, the GHS-SMOD data was used to define the spatial extent of each urban area, for which index values were calculated. In defining urban extents, the intention was to ensure that the full urban area was included, as opposed to accurately delimiting the urban area. SMOD class values of 21–30 (peri-urban, semi-dense urban cluster, dense urban cluster, urban centre) representing urban/peri-urban settlement types were all considered to be urban, and the grid cells corresponding to these classes were reclassified to create a binary urban/not-urban raster. All grid cells in the binary urban raster that were spatially contiguous with an urban centre, were considered to be part of the urban area surrounding the urban centres. Given the relatively coarse spatial resolution of the GHS-SMOD data (1 km) and the potential growth in urban settlements since 2015, the spatially contiguous urban grid cells were buffered by 3 km to try and ensure the full urban area was included. A distance of 3 km was chosen after testing a range of buffer distances and visually assessing the buffered area against the urban extent visible in recent satellite imagery. Finally, a convex hull was created around each location of contiguous urban grid cells to create a smooth polygon boundary around the buffered grid cells. If convex hulls spatially overlapped, the urban areas within these convex hulls were considered to be a single urban area, and a new convex hull was created around the combined urban areas. In the case of urban areas being near national boundaries, national administrative boundaries were used to spatially clip the urban extents. Similarly for urban areas in coastal locations, urban extents were clipped to the coastline. In total, for the 50 countries in sub-Saharan Africa covered by this dataset, there were 1,373 urban extent polygons covering an area of 239,050 km^2^, associated with 1,551 named locations in the GHS-UCDB dataset.

### Defining spatial units within urban extents

**Fig. 2:**
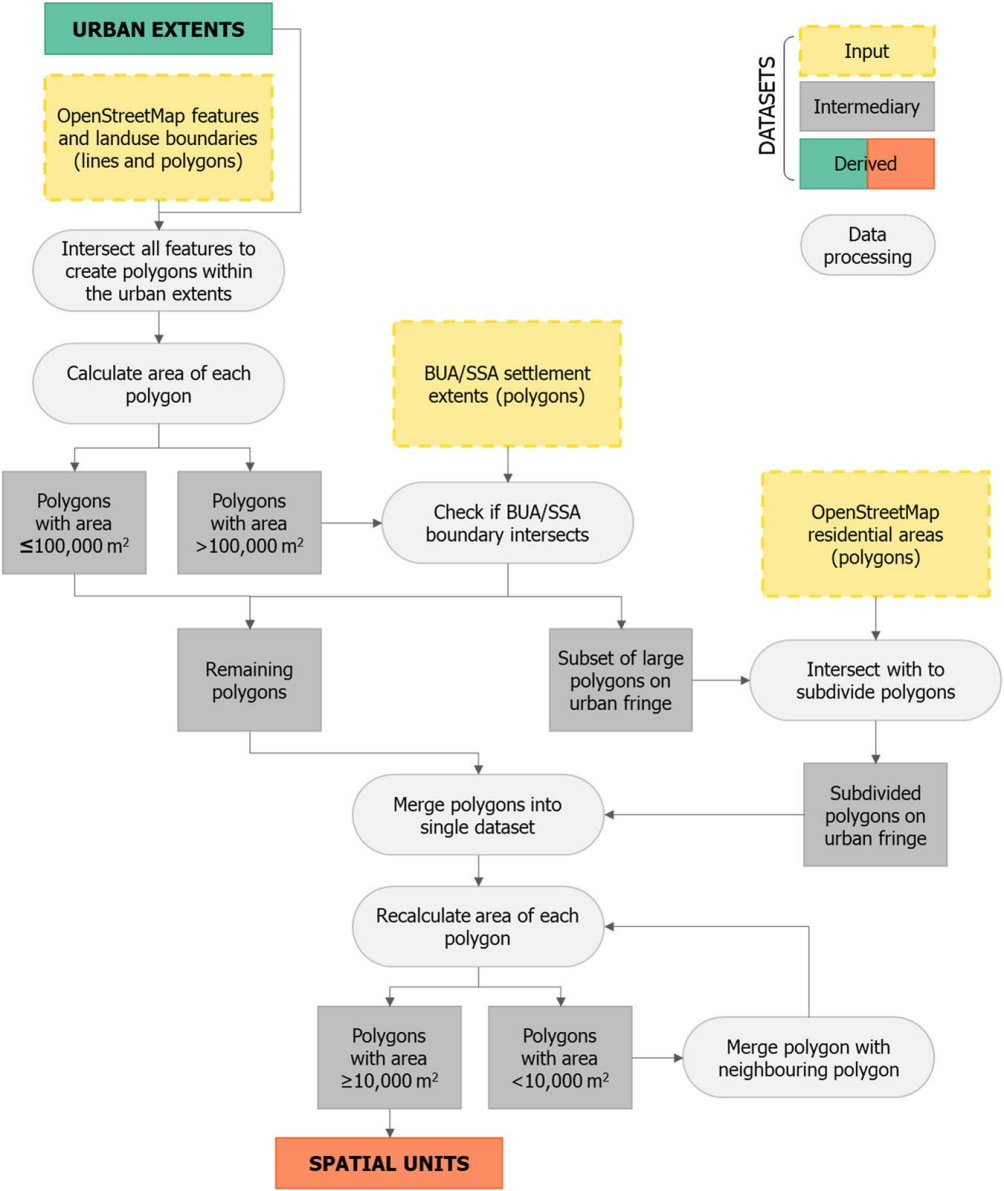
A schematic representation of the data processing steps involved in the creation of the spatial units for which the index is ultimately calculated.

As the social distancing index dataset covers 50 countries, no sufficiently small administrative or statistical unit existed across all countries to be the unit of analysis within urban areas. Instead, small spatial units were created within each urban extent (Fig. 2), for which index values were calculated. The boundaries of the custom spatial units were defined by recognisable features as far as possible, such as roads, rivers and railways. Data on these linear features were supplemented by additional line and polygon features, such as the boundaries of land use types (e.g. military areas, airports, hospital grounds, golf courses). Data on all features used in defining the boundaries of the custom spatial units was extracted from OpenStreetMap^17^ and is detailed in Table 1. Similar approaches utilising OpenStreetMap (OSM) data to create spatial units based on recognisable features have been used in sub-Saharan African cities for studies on urban land use classification^18,19^, slum mapping^20^ and semi-automated approaches to create census enumeration units^21^.

**Table 1:** The input datasets used in creating the mapped index outputs for urban areas in sub-Saharan Africa, including the data file format, purpose, source and description.

Data/*Format*	Purpose	Source/Description of dataset	Details of dataset
Urban centres/*Point (vector)*	Defining urban extents	JRC Global Human Settlement Layer - A global dataset of urban centres, represented as points	GHS (Global Human Settlement) Urban Centre Database 2015 v1.2^16,53^
Settlement types/*Gridded (raster)*	Defining urban extents	JRC Global Human Settlement Layer - A gridded dataset of settlement types at a spatial resolution of 30 arc seconds, with 8 classes (urban centre, dense urban cluster, semi-dense urban cluster, suburban/ peri-urban, rural cluster, low density rural very low density rural and water)	GHS (Global Human Settlement) - SMOD v2.0 (Settlement Model grid)^15,53^
National administrative boundaries/*Polygon (vector)*	Clipping urban extents	CIESIN - Polygons of national boundaries (administrative level 0), used in defining the extent of the gridded population datasets	National administrative boundaries, from WorldPop and Center for International Earth Science Information Network (CIESIN), Columbia University^54^
Coastlines/*Line (vector)*	Clipping urban extents	OpenStreetMap - Lines representing coastlines	Coastline features from OpenStreetMap: downloaded from https://osmdata.openstreetmap.de/data/coastlines.html
Linear and boundary features/*Polygon/line (vector)*	Defining spatial units for the index	OpenStreetMap - Polygon and line datasets representing features of the built environment and landuse boundaries	Features from OpenStreetMap^17^: - Roads, Railways, Waterways, Industrial, Cemetery, Military, Golf course, Park, Wetland, Water, Quarry, University, Hospital grounds, Reservoir, Residential - from geofabrik.de - Mine, Airport, Landfill, Basin - from QuickOSM^55^ QGIS^56^ plugin
Settlement extents/*Polygon (vector)*	Defining spatial units for the index	CIESIN GRID3 - Polygons representing settlement extents, based on grouping of building footprints. Settlement extents are classified as built-up areas (BUAs), small settled areas (SSAs) or hamlets.	GRID3 Settlement Extents version 01, alpha^22^* *Full details of each national dataset is provided in Appendix C of the Ease of Social Distancing dataset release statement^11^
Population/*Gridded (raster)*	Index calculation	WorldPop - Gridded datasets of estimated population counts at a spatial resolution of 3 arc seconds (0.0008333333 decimal degrees or approximately 100 m at the Equator). Available as national datasets.	Gridded population estimates, derived from projected population counts for 2020 produced using top-down disaggregation methods and constrained to settled grid cells^57^ with the exception of: - Sierra Leone^58^, Mozambique^59^, Zambia^60^, Burkina Faso^61^, Ghana^62^, Nigeria^63^, South Sudan^64^, Democratic Republic of the Congo^65^* *The DRC bottom-up estimates only cover 5 provinces in the west of DRC. Top-down population estimates^57^ *were used for the remaining 21 provinces*
Grid cell surface area/*Gridded (raster)*	Index calculation (to convert counts to density)	WorldPop - Gridded dataset (global coverage) with values as the surface area of each grid cell	Grid cell surface area dataset from WorldPop and Center for International Earth Science Information Network (CIESIN), Columbia University^54^
Building footprints/*Polygon (vector)*	Index calculation	Ecopia-Maxar (Digitize Africa) - Polygons of building footprints, extracted from high-resolution satellite imagery. Available as national datasets for sub-Saharan African countries	Building footprints created by Ecopia using automated feature extraction of building roofs from Maxar high-resolution satellite imagery, as part of Digitize Africa^52^

The extracted features from OpenStreetMap (OSM) were intersected to create polygons. In many locations on the urban fringe, there was a sparsity of features or land use boundaries, meaning that the polygons created by intersecting features could be quite large. To address this, further efforts were made to subdivide large polygons located on the urban fringe. Settlement extents^22^, classified as either BUAs (built-up areas) or SSAs (small settled areas), were used to identify locations on the urban fringe. Polygons which intersected the boundary of a BUA or SSA settlement extent, and were greater than 100,000 m^2^ in area, were considered to be large polygons on the urban fringe which should be further subdivided. For these selected polygons, if OSM data for residential areas was available, these were included as additional features to subdivide the existing polygons. The area of every polygon was then calculated. A minimum area constraint of 10,000 m^2^ (1 hectare) was applied; any polygons less than 1 hectare in area were merged with neighbouring polygons iteratively, until the area constraint was met. The resulting polygons were the spatial units of analysis for which the social distancing index is calculated, and are most representative of single street blocks, or groups of street blocks, in the urban centre.

If an urban extent had fewer than 30 spatial units, the urban extent was excluded from the output dataset as it was considered to most likely not actually constitute part of an urban area. Such instances either occurred along national boundaries, where an urban extent polygon spanned the national boundary but significant urban settlement was only present in one country. Alternatively urban extents with less than 30 spatial units occurred where an urban centre was very small, and insufficient features in OpenStreetMap meant it was not possible to define suitable spatial units within the urban extent. A threshold of 30 spatial units was chosen following testing of a range of threshold values.

### Calculating population density and urban form metrics

Calculating estimates of population density requires data on the geographic distribution of population. Typically, population distribution data provides a static representation of population, for example population counts derived from censuses are based on residential address locations. The spatial distribution of population though is not static, and changes constantly as people go about their daily lives. Although census data are generally considered to be the standard source for population counts, the necessity of enumerating the population at their residential location means that the population distribution derived from census data is inherently more representative of the population at night, than during the day^23^. During the daytime, populations are likely to be at school or work, running errands or undertaking other activities outside their residential location, altering the spatial distribution of population. During the COVID-19 pandemic, changes in population mobility and implementation of measures intended to reduce disease transmission, such as school closures, requirements to work from home and curfews, have also affected the spatial distribution of population^24,25^.

High-resolution data on population distributions is most commonly derived from censuses and therefore represent residential populations (e.g. gridded datasets from WorldPop). Data on other population distributions are available, although these vary in terms of their spatial coverage and resolution. Oak Ridge National Laboratory’s LandScan Global dataset^26^ represents ambient population at 1 km spatial resolution globally, LandScan USA^27^ provides daytime and nighttime population distributions for the USA, while Pop247^23^ provides time-specific population distributions for England. Considerations around social distancing feasibility are not limited to residential settings, with social distancing a necessity in a wide-range of settings where other transmission-mitigating measures are not in place. However, given the availability of data on population distributions with suitably high spatial resolution, and the general lack of additional transmission-mitigating measures in residential settings, this work focuses on social distancing feasibility considering residential population density.

**Fig. 3:**
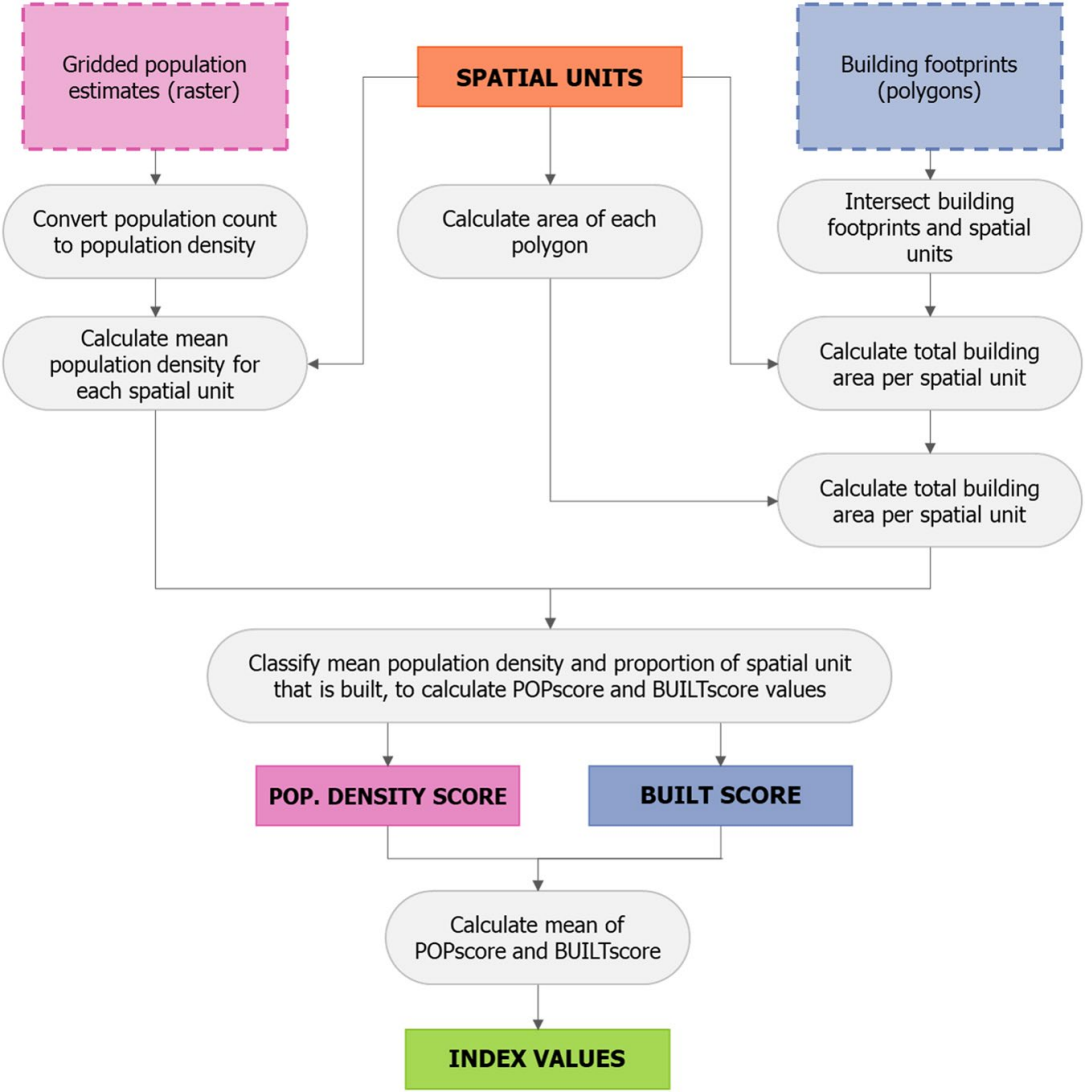
A schematic representation of the data processing steps involved in the calculation of the built and population density scores, and the subsequent calculation of the ease of social distancing index values for each spatial unit.

The ease of social distancing index is calculated from metrics of residential population density and space occupied by buildings, calculated for each spatial unit (Fig. 3). Residential population density was calculated from WorldPop high-resolution gridded population count datasets^28^ with a spatial resolution of 3 arc seconds (approximately 100 m at the Equator). There are other gridded population datasets available with similar or finer spatial resolutions (e.g. GHS-POP or HRSL)^29^, however only WorldPop population datasets are constrained to grid cells with Ecopia building footprints. As the index relies on measures of both population density and space occupied by buildings (calculated from Ecopia building footprints), it made most sense to use gridded population estimates that were spatially constrained based on the same building footprint data. WorldPop gridded population datasets are created primarily with two approaches: top-down and bottom-up estimation modelling^30^. The top-down approach creates gridded estimates through the spatial disaggregation of enumerated census population counts or projections for administrative units. In contrast, the bottom-up approach takes enumerated population counts for small areas, for example from household survey listings, and uses a geostatistical model to estimate population for all grid cells, including in unsampled locations^30^. Particularly for locations where population projections are uncertain due to a long time period having elapsed since the last census, bottom-up modelled population estimates are likely to provide a more realistic estimate of population counts and spatial distribution. For this reason, where WorldPop bottom-up population estimates are available, these have been used as the data source for the population density component of the ease of social distancing index. For countries where bottom-up estimates were not available, gridded population estimates produced using a top-down constrained approach were used instead.

The WorldPop top-down approach involves dasymetric modelling of census counts or projections, using a Random Forest model to estimate a weighting layer from a range of ancillary gridded covariates^31^. In the “constrained” approach, a binary settlement mask is used to constrain the disaggregation to grid cells identified as having one or more buildings present. In the case of top-down constrained population datasets for sub-Saharan African countries, the binary settlement mask is based on Ecopia building footprints. Ancillary covariates derived from the building footprints, such as building count, building area, variation in building area and distance to edge of settled area are also included as inputs to the Random Forest model (used to estimate the dasymetric weighting layer). Further details of the gridded population datasets are provided in Table 1.

To calculate a mean population density for each spatial unit, a population density raster was first created for each country by dividing the grid cell values in the population count raster, by the surface area for each grid cell, maintaining a spatial resolution of 3 arc seconds. From the density raster, an estimate of mean population density was then calculated for each spatial unit, using zonal statistics. In the event that a spatial unit was of a size or shape such that there were no grid cell centroids of the population raster located within it, a mean zonal statistic could not be calculated. Instead, for the centroid of the spatial unit, an estimate of mean population density was calculated by interpolating neighbouring grid cell values. For spatial units in close proximity to a national boundary, there were some locations where it was not possible to calculate a mean population density, due to differences in the spatial extents of datasets resulting in missing population estimates. Where this occurred, spatial units have been assigned a no data value (−99) for the population density score.

The second metric used in the index was a measure of space occupied by buildings, calculated from building footprint polygons. Developments in computing power and the increasing availability of very high-resolution satellite imagery, have enabled new datasets of building footprint polygons to be produced using feature extraction techniques. For sub-Saharan Africa, existing building footprint datasets include those from Microsoft (https://www.microsoft.com/en-us/maps/building-footprints), Google (https://sites.research.google/open-buildings) and Ecopia (https://www.ecopiatech.com/global-feature-extraction). The differences between these datasets, and the extent to which they can be used interchangeably has not been systematically explored and this will be a focus of future work. Only the Ecopia building footprints have coverage for all countries in sub-Saharan Africa and hence were used in calculating the built metric of the social distancing index.

For each spatial unit, the proportion of space occupied by buildings was calculated by summing the total building footprint area, divided by the area of the spatial unit. In instances where building footprints spanned more than one polygon, the building footprint polygon was split into two or more smaller polygons along the boundary of the spatial unit. This metric was solely derived from building footprint data and does not consider building height or associated floor space. To incorporate measures of available floor space would require detailed building height data for all cities across sub-Saharan Africa, which was not available. In assessing social distancing feasibility, estimating available floor space is important in areas where high population densities are associated with high-rise buildings which have a relatively small building footprint. In the context of sub-Saharan African urban areas, the prevalence of high-rise buildings varies but they are typically not the norm for residential dwellings. More commonly high population densities are associated with informal settlement areas^5^, where building height is commonly one- or two-storeys. Whilst data on building height were not available to incorporate into the index, a new urban morphological dataset^32^, available for major cities globally, provides a classification of urban form which considers building height. Comparing the ease of social distancing index values with this urban morphological classification for 12 cities (Supplementary Information Fig. 1), indicates that for the majority of cities, areas of high- and mid-rise buildings are not associated with high index values.

**Fig. 4:**
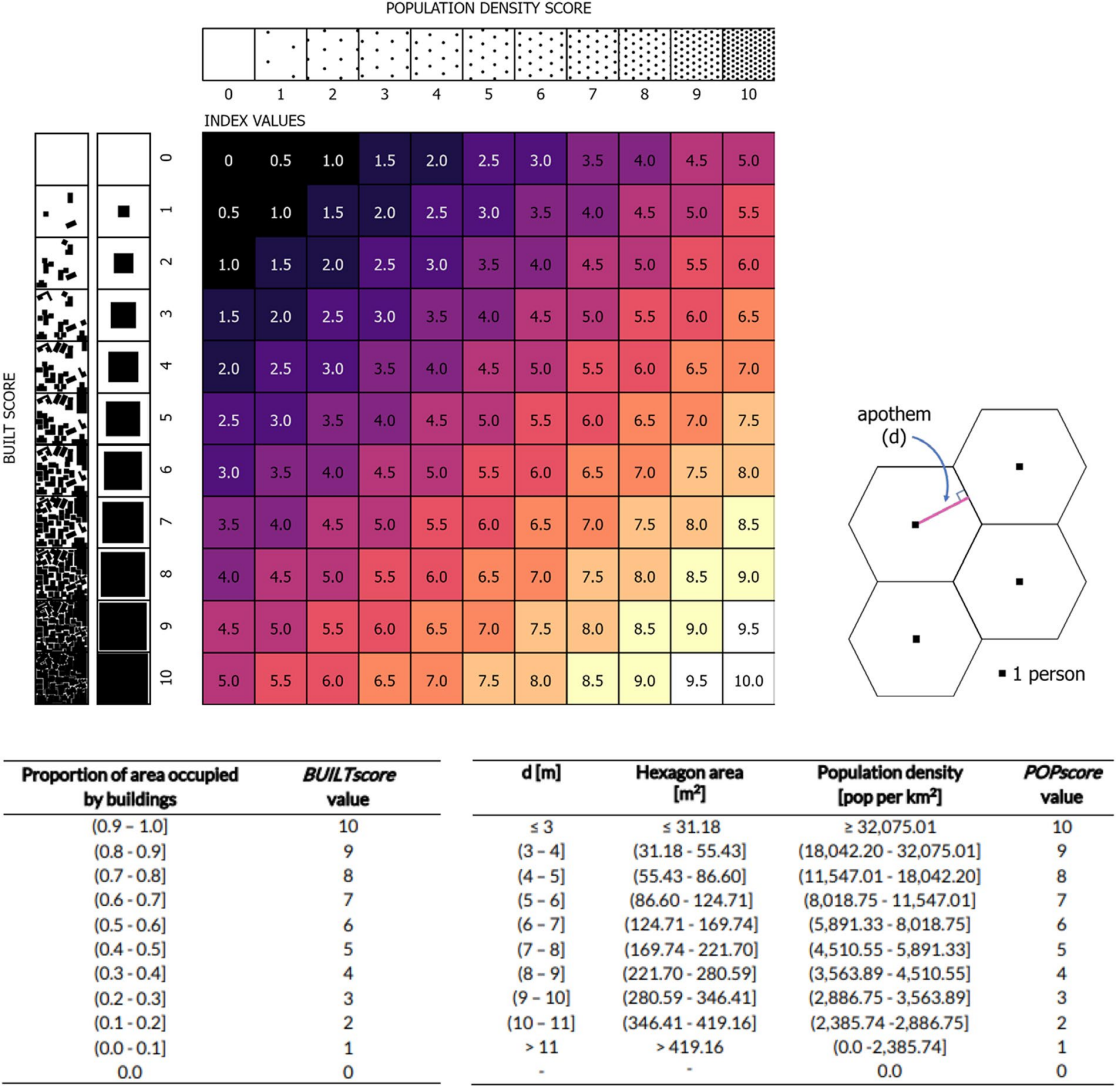
The population density and built scores are evenly weighted in the index, calculated as the mean of the two scores (top). Examples of the spacing of buildings and population for each score value is included, with the built score shown with building footprints representing both a single and multitude of buildings. The population density score is calculated based on a hexagon tessellation with a range of distancing parameters, with the classification of built and population scores provided (bottom).

To convert the estimates of mean population density and proportion of space occupied by buildings into scores, the estimates were then classified with values assigned between 1 and 10. Whilst this is an arbitrary and subjective choice of classes, the values are easy to interpret and it provides a level of detail that adequately distinguishes between different levels. Values for the proportion of space occupied by buildings in a spatial unit were assigned values between 1 and 10; spatial units in which 10% or less of the space was occupied by buildings were assigned a value of 1, greater than 10% but less than or equal to 20% were assigned a value of 2. Classified values were assigned in this linear fashion up to a value of 10, which was assigned where over 90% of a spatial unit was occupied by buildings. If a spatial unit had no building footprints within it, then a value of 0 was assigned (Fig. 4).

Mean population density values were classified based on the population density that is possible with different distancing parameters. To estimate population density with a range of distancing parameters, an idealised model of perfect spacing between people, based on a hexagon tessellation was used. In this conceptualisation, the hexagon represents the space available for one person, with the person considered as the hexagon centroid (Fig. 4). The distance between the centroid of a hexagon and the midpoint of a side is termed the apothem (d), with the distance between centroids of neighbouring polygons being twice the apothem length. Considering a social distancing requirement of 2 m distance, for all individuals in a spatial unit to maintain 2 m from each other, whilst being able to move, an apothem of 3 m is required. This distance ensures that a person can move 2 m in any direction, whilst still maintaining a distance of 2 m from any other person. The area of a hexagon with an apothem of 3 m (i.e. the area needed for one person to maintain 2 m distance whilst being able to move) is 31.18 m^2^, which corresponds to a population density of 32,075 people per km^2^. Spatial units with a mean population density of greater than or equal to 32,075 people per km^2^ are assigned a value of 10 - the maximum population density score.

Population density scores were assigned to the remaining population density estimates using threshold values derived for increased spacing between people. The hexagon apothem was increased in increments of 1 m, up to 11 m. A hexagon with an apothem of 11 m covers an area of 419.16 m^2^ for which the corresponding maximum population density is 2,386 people per km^2^. A value of 1 was assigned to spatial units with non-zero population density estimates less than 2,386 people per km^2^. The population density thresholds are outlined in Fig. 4. Any spatial unit with 0 estimated population was assigned a value of 0. The calculation of population density thresholds are theoretical and do not account for obstructions which may be present and affect the space available for individuals to move within and distance from each other. The distances used to calculate population density thresholds will in reality most likely be reduced, due to the space within built environments rarely being open and free from obstructions.

### Calculating the social distancing index

**Fig. 5:**
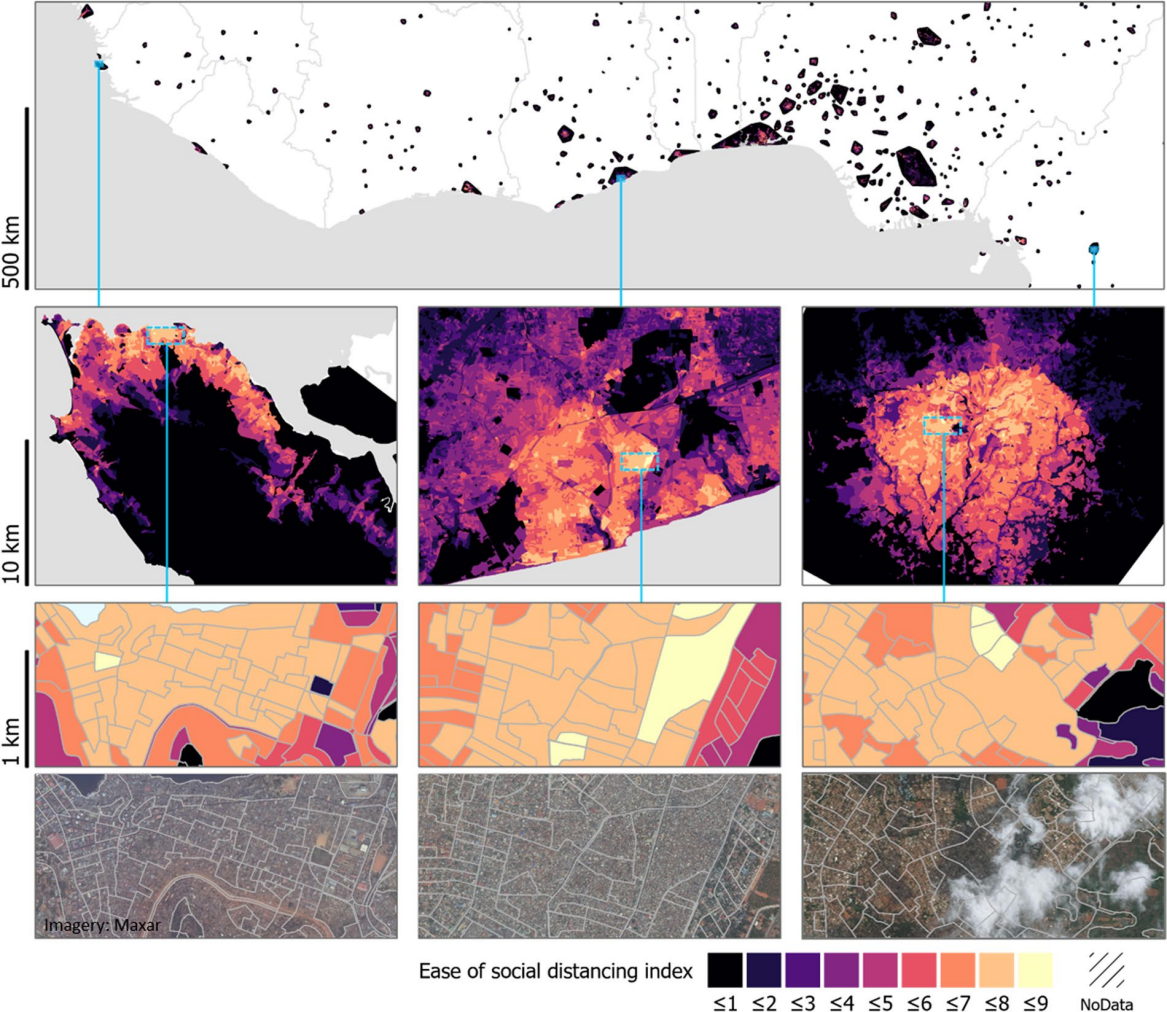
Mapped ease of social distancing index outputs are shown for West Africa, with examples for Freetown, Sierra Leone (left column), Accra, Ghana (centre column) and Yaoundé, Cameroon (right column). The lower panes show larger-scale maps (middle rows) with examples for locations with higher index values, shown both with the mapped index values and the spatial units overlaid with Maxar satellite imagery from 2018–2020 (bottom row). The higher index values (paler colours) indicate greater expected difficulty in practising social distancing.

The index value for each spatial unit was calculated as the mean value of the population and built scores, resulting in index values ranging from 0 to 10. An index value of 10 would indicate a very high population density (≥32,075 population per km^2^) and a very high density of buildings (>90% of spatial unit area is occupied by buildings); a combination of factors which would mean that social distancing is extremely difficult. In contrast, an index value of 1 would indicate a relatively low population density and considerable space available around buildings, likely associated with fewer physical constraints for social distancing. A value of 0 indicates that a spatial unit has no buildings and no estimated population, and consequently no social distancing constraints are anticipated. A no data value (−99) was assigned for any spatial units which had a no data value for the population score. Mapped index values for West Africa are shown in Fig. 5, with detailed maps included for the capital cities of Sierra Leone, Ghana and Cameroon.

## Data Records

The ease of social distancing index datasets^11,12^ for urban areas in 50 sub-Saharan African countries are openly available to download from the WorldPop Open Population Repository (https://wopr.worldpop.org/?/SocialDistancing) in Shapefile format. To download files for multiple countries at once, we recommend using the WOPR API or wopr R package (see relevant tabs at https://apps.worldpop.org/woprVision/ for guidance). The datasets can also be viewed interactively through the GRID3 Data Hub (https://data.grid3.org/). Further details of the datasets, including a description of each field, are outlined in Table 2.

**Table 2:** Details of the output data files of the ease of social distancing index.

File/description	Variables
XXX_SocialDistancing_v1_0_index Dataset of spatial units (polygons) within urban areas, with index values calculated for each spatial unit. The population density and built score values are also included as attributes^11,12^	uext_ID: ID of the urban extent. Common ID which can be used to link with the XXX_SocialDistancing_v1_0_urban_extents and XXX_SocialDistancing_v1_0_urban_points files.
	adm0_ISO3: The alpha-3 letter ISO-3166 code of the country dataset
	UNIT_AREA: The area of the spatial unit [metres squared]
	BUILT_AREA: The summed area of all building footprint polygons within the spatial unit [metres squared]
	BUILT_PROP: The proportion of the spatial unit that is built, calculated as BUILT_AREA/UNIT_AREA
	NBUILTPROP: The proportion of the spatial unit that is not built, calculated as 1-BUILT_PROP
	POP_DENS: The mean population density (population per kilometres squared) of the spatial unit, calculated for all grid cells that have their centroid within the spatial unit
	BUILTscore: Built score (0–10), classified based on the BUILTPROP field. A value of 10 corresponds to over 90% of the unit area being occupied by buildings. A value of 1 corresponds to less than 10% of the unit area being occupied by buildings, but with at least 1 building present. A value of 0 indicates a spatial unit with no buildings present.
	POPscore: Population density score (0–10), classified based on the POP_DENS field. A value of 1 corresponds to low population density and a value of 10 corresponds to very high population density. A value of 0 indicates a spatial unit where the mean population density is 0 people per kilometre squared. A no data value (−99) indicates missing data.
	INDEXvalue: Ease of social distancing index value (0–10), calculated as the mean of the BUILTscore and POPscore field values. A value of 1 is indicative of relative ease of social distancing due to low population density and ample space around buildings. A value of 10 is indicative of high difficulty in maintaining social distancing due to very high population density and very little space around buildings. A no data value (−99) indicates missing data.
XXX_SocialDistancing_v1_0_urban_extents The urban extents within which spatial units are defined, and for which index values are available^11,12^	uext_ID: ID of the urban extent. Common ID which can be used to link with the XXX_SocialDistancing_v1_0_urban_extents and XXX_SocialDistancing_v1_0_urban_points files.
	adm0_ISO3: The alpha-3 letter ISO-3166 code of the country dataset
XXX_SocialDistancing_v1_0_urban_points Points representing urban centres, extracted from the GHS Urban Centre Database 2015 v1.2. Names of urban centres are included as attributes^11,12^	PNT_LAT: Latitude of urban centre point location (decimal degrees)
	PNT_LON: Longitude of urban centre point location (decimal degrees)
	adm0_NAME: Name of country
	adm0_ISO3: The alpha-3 letter ISO-3166 code of the country dataset
	urb_NAME: Name of urban centre
	uext_ID: ID of the urban extent. Common ID which can be used to link with the XXX_SocialDistancing_v1_0_urban_extents and XXX_SocialDistancing_v1_0_urban_points files.

Files are available for each country, identified by the first three letters in the file name (XXX) being the alpha-3 letter ISO-3166 code of the country dataset. A full list of ISO codes and corresponding country names is available in Appendix 1 of the dataset release statement.

## Technical Validation

The ease of social distancing index values are calculated from metrics derived from gridded population estimates and building footprints. These input datasets were already checked by the data producers to ensure they comply with the intended quality-standards. In terms of the building footprints, the data producers implemented both automated checks and a manual review process. This includes the manual review of a randomly-selected area of 50 km^2^, for every 1000 km^2^ of processed imagery^33^, and checks to ensure that the dataset meets specified quality requirements (≥90% valid interpretation)^34^. Gridded population datasets are harder to validate, particularly at fine spatial resolutions or at the grid-cell level^29^. Gridded population datasets for sub-Saharan African countries, produced using the WorldPop top-down constrained approach, utilise the best-available census-derived data on population totals, carefully selected geospatial covariates and building footprint datasets. Output datasets are checked to ensure that grid cell values sum to the administrative unit totals used as input for the dasymetric modelling, have a sufficiently high explained variance (e.g. >0.8) and spot-checked through visual comparison with satellite imagery basemaps. For WorldPop gridded population datasets produced using bottom-up modelling approaches, similar geospatial covariates and building footprint datasets are used, with high-quality population enumeration datasets, typically collected in partnership with national statistical offices. The resulting gridded population datasets include uncertainty estimates at the grid cell level, and for aggregated totals. A review process involving calculating various goodness of fit metrics, cross-validation, comparison of the outputs with alternative population data sources and recent high-resolution satellite imagery is undertaken. Where possible the estimates are also reviewed by staff from national statistical offices. In the context of this work, additional visual checks of mapped input datasets were implemented. For the resulting index values, mapped outputs were checked on a country-by-country basis, and the statistical distributions of index values reviewed. Any issues identified in this process are listed in the data release statement Appendix E^11,12^.

It is difficult to validate if the ease of social distancing index values reflect the reality of social distancing feasibility within and between urban areas. However, in the context of urban sub-Saharan Africa, populations in slums and/or informal settlements are recognised as experiencing challenges in social distancing^2–6^. Consequently, we would expect that high index values would be associated with these types of settlements. UN-Habitat characterises informal settlements as residential areas where inhabitants do not have security of tenure, often have limited access to basic services and infrastructure, with housing that may not meet relevant building and planning regulations, and may be located in environmentally-hazardous locations^35^. However, informal settlements take many forms^36^ and there is no accepted formal definition of slums or information settlements^37^. Locations of informal settlements and slums are also not routinely mapped in a standardised way, and are often home to populations that can be excluded from official statistics^38,39^.

**Fig. 6:**
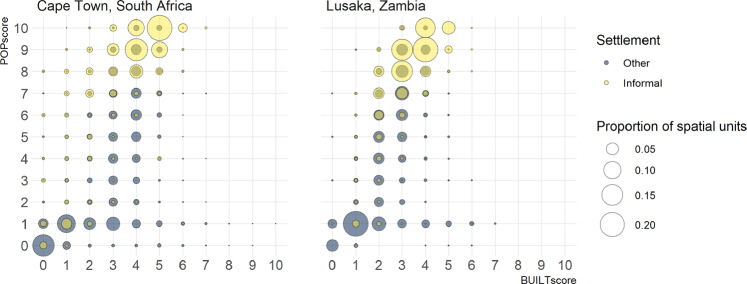
Considering all spatial units within the urban extents of (**a**) Cape Town, South Africa and (**b**) Lusaka, Zambia, the population density score (POPscore) is plotted against the built score (BUILTscore). Spatial units are classified according to settlement type, as either being within an informal settlement (yellow) or not, i.e. all other locations (blue). The size of the circle denotes the proportion of spatial units with each combination of POPscore and BUILTscore values in the two settlement types.

Mapped datasets of informal settlements do exist for some cities, for example informal settlements in Cape Town, South Africa are available as mapped extent polygons (https://africaopendata.org/dataset/city-of-cape-town-gis-data). Recent work by the International Growth Centre (IGC) with Ordnance Survey (OS), characterised informal settlements in Lusaka, Zambia, with detailed maps of settlement types^40^. These spatial datasets provide mapped locations of informal settlements, but are limited in their coverage to individual cities and cannot be assumed to be consistent in the definition used in mapping informal settlements. The ease of social distancing index values, and the associated population density and built scores, were compared with the maps of informal settlements in Lusaka and Cape Town (Fig. 6). Spatial units in these cities were classified as one of two settlement types: informal settlements or other (not informal settlements). For Lusaka, the IGC/OS detailed maps of settlement types provided information at the building level; the majority settlement type for each spatial unit was determined and reclassified to the binary informal settlement classes. In contrast, the informal settlement dataset for Cape Town was less spatially-detailed and consisted of polygons representing informal settlement extents. We took a conservative approach and considered any spatial unit with 50% or more of its area within a settlement polygon as being an informal settlement. For spatial units within the areas considered to be informal settlements (“Informal”), the proportion of spatial units with each combination of population density and built scores was calculated, and compared against those spatial units in the areas that are not classified as informal settlements (“Other”) within the same spatial extent. An additional comparison with areas classified as planned residential in Lusaka, is shown in Supplementary fig. 2.

Figure 6 shows that a greater proportion of the spatial units in areas of informal settlement had higher population density and built score values, than spatial units in other types of settlement. For Lusaka, 92.3% of spatial units in informal settlements had a population density score of 7 or greater, while for other settlement types, only 35.8% of spatial units did. Similarly for Cape Town, 84.9% of spatial units in informal settlements had a population density score of 7 or greater, while for other settlement types, only 20.4% of spatial units did. Focussing on the highest population density scores, 58.8% and 66.7% of spatial units in informal settlements in Lusaka and Cape Town respectively, had a population density score of 9 or greater with the same score only for 16.6% (Lusaka) and 7.4% (Cape Town) of spatial units in other settlement types. Built score values are consistently lower than population density score values, but higher values are still observed for spatial units within informal settlements than other settlement types. 45.4% of spatial units within informal settlements in Lusaka and 69.7% of spatial units within informal settlements in Cape Town had a built score value greater than or equal to 4. In comparison, only 18.9% and 31.6% of spatial units in other settlement types in Lusaka and Cape Town respectively had a built score value greater than or equal to 4. The higher score values found in informal settlement locations are reflected in the index values also, with 61.7%/73.4% of spatial units in informal settlements (Lusaka/Cape Town) having an index value of 6 or greater, compared to 19.3%/12.0% of spatial units in other settlement types (Lusaka/Cape Town). Examining index scores for areas of informal settlements in Lusaka and Cape Town, confirms that high index values, indicating greater difficulty in social distancing, are found in locations where social distancing is recognised as being more difficult. This comparison would ideally be repeated across all urban extents in the ease of social distancing index dataset, but this is not possible given the limited mapped data on informal settlement extents.

Gibson and Rush^10^ assessed social distancing feasibility by calculating the distance between buildings in two informal settlements in Cape Town. In both settlements: Masiphumelele and Klipfontein Glebe, it was identified that social distancing would be very difficult given the dense arrangement of buildings^10^. Settlements with these names are included in the Cape Town informal settlement dataset described previously, however the spatial extents of the settlements differ and only overlap for the settlement of Masiphumelele. The ease of social distancing index values for spatial units in Masiphumelele are generally high (minimum: 8, maximum: 8.5); the index values are in agreement with Gibson and Rush’s findings for Masiphumelele. For the settlement of Klipfontein Glebe only a qualitative assessment was carried out due to the spatial mismatch in the area identified as Klipfontein Glebe. The area considered to be Klipfontein Glebe in Gibson and Rush’s work did not have particularly high index values. The low index values for this area are driven by low population density scores, whilst built scores remain relatively high. Reviewing the values of the gridded population dataset for Klipfontein Glebe shows surprisingly low values given the number and density of built structures. The gridded population estimates used in calculating index values for South Africa are top-down estimates (i.e. disaggregated from census projections). The discrepancy between the population score and the built score may be the result of a potential under-enumeration of the population in this area during the census or growth of settlement since the last census. This is consistent with the findings of Thomson *et al*.^41^ who identified underestimates of population in gridded datasets for slum areas in Nigeria and Kenya. Future work to improve the accuracy of gridded population estimates in informal settlements, such as the development of bottom-up modelled estimates^42,43^, will likely benefit derived datasets such as the ease of social distancing index.

## Usage Notes

The ease of social distancing index datasets provide estimates of social distancing feasibility for urban areas across sub-Saharan Africa. The index values are calculated for small spatial units, providing mapped estimates at high spatial resolution. The datasets are available to download in Shapefile format and users can work with these in Geographic Information Systems (GIS) software or other software with spatial analysis capabilities. These datasets can support a range of applications, both directly associated with COVID-19 response and more broadly related to public health, urban planning and accessibility. For example, an analysis to identify locations with poor access to COVID-19 testing, could also consider where social distancing is most difficult as locations with both poor access to testing and difficulty in social distancing may be susceptible to rapid community transmission that is not detected through standard testing programmes. In such locations, infected individuals are unlikely to be able to effectively self-isolate at home and therefore to prevent further community transmission, support and provision of accessible facilities nearby to self-isolate are likely needed^5^. Given potential differences in testing- or treatment-seeking behaviour, the ease of social distancing index may be beneficial in planning sampling frames for COVID-19 seroprevalence surveys in terms of stratification by neighbourhoods or settlement types. The index datasets may also have a role in community advocacy, for example in providing quantitative data and mapped outputs to communities experiencing difficulties in social distancing and associated challenges with overcrowding. Aside from the COVID-19 pandemic, the ease of social distancing index can be useful in identifying locations susceptible to other risks. Locations with high population densities and overcrowding, particularly when combined with poor ventilation, can provide conditions favourable for transmission of other airborne infectious diseases, such as influenza, tuberculosis or measles^44^. Other physical hazards are also a concern in such locations; for example, buildings in close proximity and constructed from flammable materials are at particular risk of rapid fire spread - conditions that are commonly found within informal settlements^10^.

The following limitations have been identified with the ease of social distancing datasets. Firstly, the index is calculated from data on urban form and population density to capture factors affecting social distancing feasibility at a high spatial resolution. The building footprint data were created through automated feature extraction of satellite imagery, and represent the spatial extent of buildings but do not include information on building height or use (e.g. whether the building is used for residential purposes). The imagery used in the extraction of the building footprints was acquired over multiple years. Greater than 80% of the imagery for the area covered by the ease of social distancing index is from 2018 or 2019^45^, however some imagery therefore predates 2018, and cloud cover in the satellite imagery may introduce false negatives into the building footprint datasets. The time point of the input gridded population datasets also varies. For the majority of locations where WorldPop top-down population datasets based on projected census figures were used, the projected estimates were for 2020. For locations where other population estimate datasets have been used (Burkina Faso, Ghana, Nigeria, Mozambique, South Sudan, Sierra Leone, Zambia and the Democratic Republic of the Congo), the time point of the population estimates varies between 2015 and 2019 as estimates were only available for a single year. Population density was estimated from a single dataset for each country, however other gridded population datasets at similar spatial resolutions are available^29^. Between datasets, there can be marked variation in estimates of population at the grid cell level^29,41,46,47^. This is potentially a considerable source of uncertainty for analyses using gridded population datasets, including the ease of social distancing index. Future work will explore alternative datasets and potential methods for integrating multiple datasets.

Secondly, the gridded population datasets provide estimates of residential or nighttime population density. Given that significant diurnal population movements occur within cities, the index values may well change if estimates of daytime population density were used instead. There is however a lack of datasets representing daytime population distributions within urban areas, e.g. LandScan USA^27^, Pop247^23^ and Batista e Silva *et al*.^48^ provide daytime population estimates, but are limited to the USA, England and Europe respectively. Other data on population mobility such as call detail records (CDRs) from mobile phones or Facebook mobility data, could be used to capture these different spatial distributions where available (e.g.^24,49,50^). Population movements that occur at particular times and in particular places may also influence social distancing feasibility, for example transport hubs and markets. In these locations large numbers of people can congregate at certain times, and integrating data on population mobility could help identify such locations.

Thirdly, in addition to population density and urban form, socioeconomic factors influence social distancing feasibility, however accurate data on these factors which is georeferenced at a granular level is rarely available. Socioeconomic factors such as employment in the informal sector/reliance on daily wages, use of communal WASH facilities and dependence on crowded public transport can have a multiplicative effect in increasing difficulty in social distancing. Conversely, a secure income source from employment which it is possible to do whilst working from home, can increase the feasibility of social distancing. Such individual or community-level factors will also influence social distancing feasibility. As the index values are solely based on estimates of population density and urban form, which themselves have their own limitations, the dataset should be considered as one source to guide response efforts, but should not be relied on as the sole basis for decision-making. Finally, the ease of social distancing index dataset is limited in its spatial coverage to urban areas in 50 countries in sub-Saharan Africa. This index has been developed with these contexts in mind, but social distancing challenges will also be present in smaller towns and some rural locations within the countries covered by the dataset. Future work will look to expand the coverage of the index datasets, both outside major towns and cities, through the development of a gridded version of the index, and to a greater number of countries. We will also look to incorporate additional factors, for example data on building height, and explore alternative sources for calculating population density, including self-reported slum population values.

## Supplementary information

Supplementary Information

Supplementary Figure 1

Supplementary Figure 2

## Data Availability

The code for data processing and analysis was written in Python (version 3.6.9), using ArcPy in an ArcGIS Notebook with ArcGIS Pro (version 2.5.1)^51^. The DigitizeAfrica building footprints^52^ used in creating the output index for urban areas in sub-Saharan Africa are available for humanitarian purposes on request from Ecopia (https://www.ecopiatech.com/). Similar datasets for some countries are openly-available, such as Microsoft building footprints or Google Africa Open Buildings. The code used to create the spatial units and calculate the index values described in this paper is available to download from GitHub, in the following repository: https://github.com/heatherchamberlain/SocDistIndex.
