# Supplementary material for: High-resolution estimates of social distancing feasibility, mapped for urban areas in sub-Saharan Africa
Source: Sci Data. 2022 Nov 18;9:711. doi: 10.1038/s41597-022-01799-0 (PMC9673897; doi:10.1038/s41597-022-01799-0)
Supplement: Supplementary file 1 — Supplementary Information [file 41597_2022_1799_MOESM1_ESM.pdf]

Supplementary Information for "High-resolution estimates of social distancing feasibility, mapped for urban areas in sub-Saharan Africa"

Contents

Note 1 – Comparison of index values with urban morphological classes, for selected major cities in sub-Saharan Africa. 2

Note 2 – Comparison of ease of social distancing index values, and the associated population density and built scores, with mapped settlement types in Lusaka. 4

Supplementary Information References 5

Note 1 – Comparison of index values with urban morphological classes, for selected major cities in sub-Saharan Africa.

As high-resolution data on building heights was not available for all cities in sub-Saharan Africa, it was not possible to account for building volume and associated floor space, in the ease of social distancing index. This could lead to unrealistic index values, particularly in locations where high population densities are associated with high-rise buildings. The prevalence of high-rise buildings varies considerably between cities in sub-Saharan Africa, but for most cities, they do not tend to be the norm for residential dwellings. Although data on building heights was not available when this index was developed, a new urban morphological classification[1], provides an opportunity to compare the ease of social distancing index value. The urban morphological classification, with a spatial resolution of 100m, covers major cities globally and was classified from Sentinel-1 SAR and Sentinel-2 multispectral imagery. It includes morphological classes for a range of building heights: classes 1, 2, 4 and 5 represent compact high-rise, compact mid-rise, open high-rise and open mid-rise morphology types respectively.

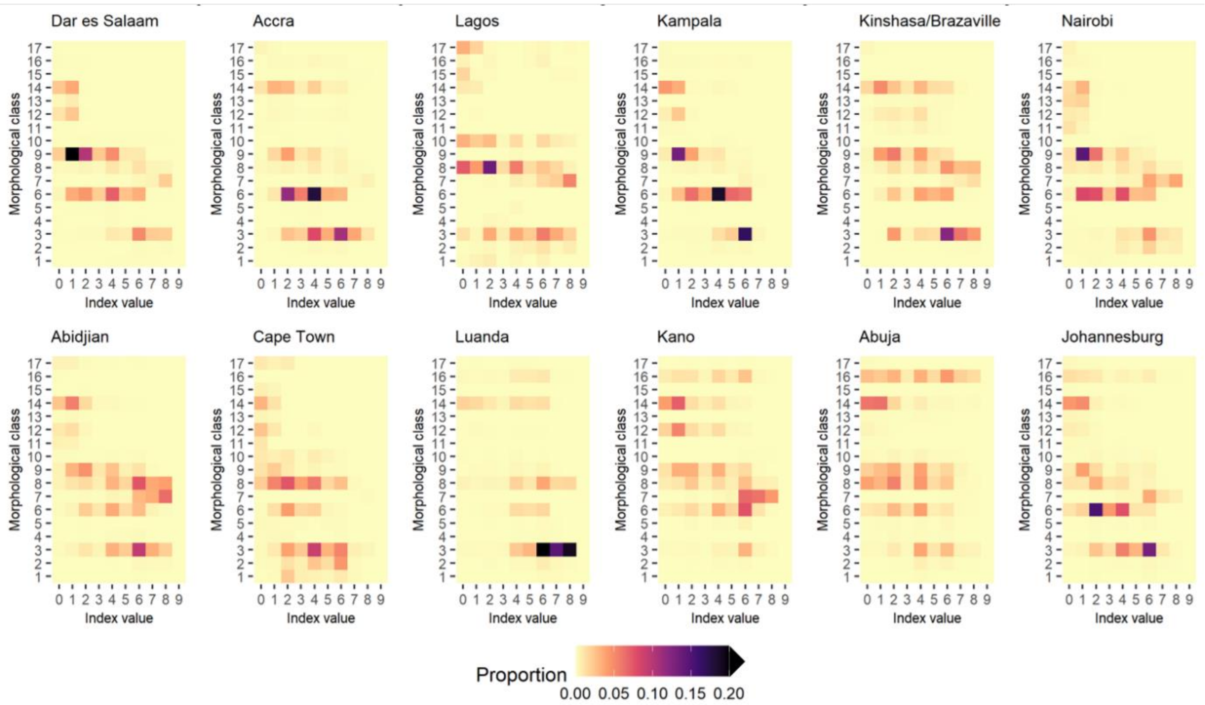

Supplementary Figure 1: *Comparison of the Ease of Social Distancing Index values[2,3] and urban morphological classes[1], for 12 cities in sub-Saharan Africa. The proportion of spatial units in each morphological class and with each aggregated index value (0-9) are shown. Locations where mid- and high-rise structures are predominant are represented by morphological classes 1 (compact high-rise), 2 (compact mid-rise), 4 (open high-rise) and 5 (open mid-rise).*

We compared the index values against the urban morphological classes for 12 major cities in sub-Saharan Africa, for the areas where both datasets were available. For each spatial unit, the urban morphological class value for that location was extracted (if more than one class was present within a single spatial unit, the majority class value was selected). The index values were rounded to convert the index values to integers (0-9). The integer index

values and urban morphological class values were cross-tabulated and the proportion of spatial units with each combination of values calculated per city (Supplementary Figure 1).

Focussing on morphological classes 1 and 2 (compact high- and mid-rise), we can see that for all cities apart from Cape Town, Nairobi and Lagos, only a small proportion of spatial units (less than 1.8%) are located in areas classified as compact high-rise or compact mid-rise. For Nairobi, Lagos and Cape Town, the proportion of spatial units located in areas classified as compact high-rise or compact mid-rise, is higher at 4.7%, 5.4% and 14.9% respectively. There are far fewer spatial units in classes 4 and 5, but overall a similar pattern is observed with the greatest proportion of spatial units located in areas classified as open high-rise or open mid-rise found in Cape Town (1.3%), and lower proportions for all other cities. In terms of the index values associated with these units, the total proportion of spatial units which have an index value of 7 or higher, and are located in areas of compact- or open- high/mid-rise (classes 1, 2, 4 or 5) is 1.0%, 0.98% and 0.38% for Nairobi, Lagos and Cape Town respectively. This comparison shows that for the majority of these cities, areas of high- and mid-rise buildings are not associated with high index values.

Note 2 – Comparison of ease of social distancing index values, and the associated population density and built scores, with mapped settlement types in Lusaka.

Figure 6 in the main text shows a comparison of index values, and the associated population density and built scores, with mapped settlement types in Lusaka and Cape Town. The data available on informal settlements for Cape Town, limited this comparison to two settlement type categories: “Informal” (informal settlements) and “Other” (all other locations). Additional data on settlement types was available for Lusaka, enabling a sub-category of “Planned residential” to be compared as shown in Supplementary figure 2. Note that the planned residential category is a subset of the “Other” category.

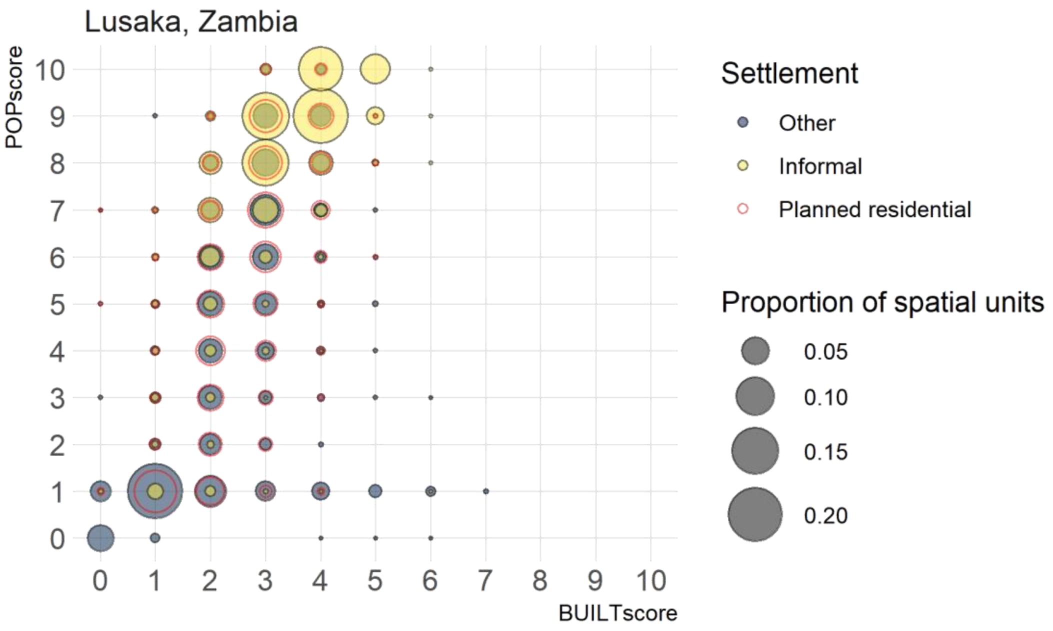

Supplementary Figure 2: *For spatial units within the urban extent of Lusaka, Zambia, the population density score (POPscore) is plotted against the built score (BUILTscore). As in Figure 6, spatial units are classified according to settlement type, as either being within an informal settlement (yellow) or not, i.e. all other locations (blue). An additional sub-category of planned residential is also included – this is a subset of the “Other” category, where spatial units are classified as planned residential. The size of the circle denotes the proportion of spatial units with each combination of POPscore and BUILTscore values in each of the settlement types.*

Supplementary Information References

1 Zhu, X. X. *et al.* The urban morphology on our planet – Global perspectives from space. *Remote Sensing of Environment* **269**, 112794, https://doi.org/10.1016/j.rse.2021.112794 (2022).

2 Chamberlain, H. R., Lazar, A. N. & Tatem, A. J. Ease of Social Distancing Index, mapped for urban areas in sub-Saharan Africa, version 1.0. WorldPop, University of Southampton. https://doi.org/10.5258/SOTON/WP00711 (2021).

3 Chamberlain, H. R., Lazar, A. N. & Tatem, A. J. Ease of Social Distancing Index, mapped for urban areas in sub-Saharan Africa (single country update for Sierra Leone), version 1.1. WorldPop, University of Southampton. https://doi.org/10.5258/SOTON/WP00721 (2021).
