# Supplementary figures and images for: High-resolution estimates of social distancing feasibility, mapped for urban areas in sub-Saharan Africa
Source: Sci Data. 2022 Nov 18;9:711. doi: 10.1038/s41597-022-01799-0 (PMC9673897; doi:10.1038/s41597-022-01799-0)

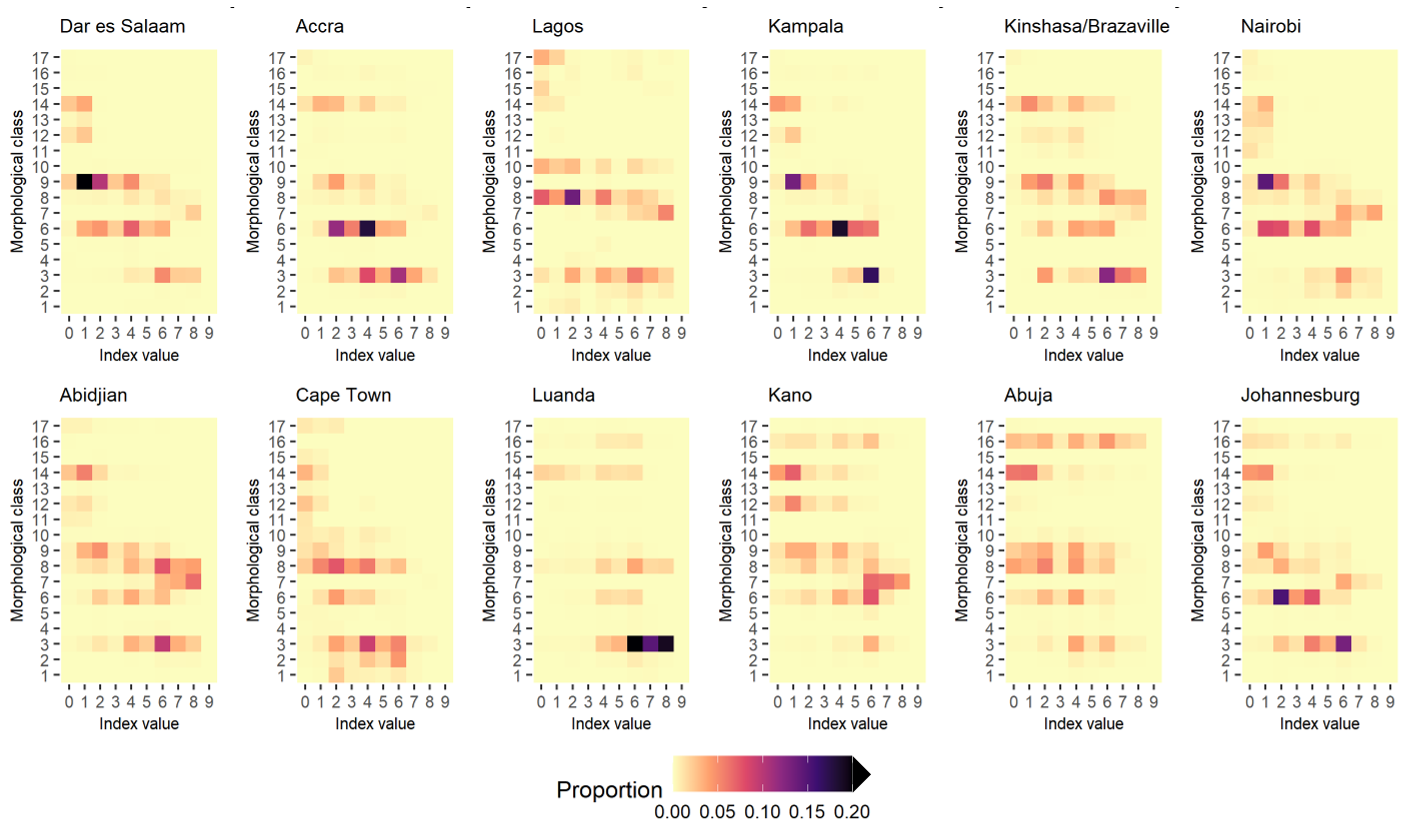

Supplement: Supplementary file 2 — Supplementary Figure 1 [file 41597_2022_1799_MOESM2_ESM.png]

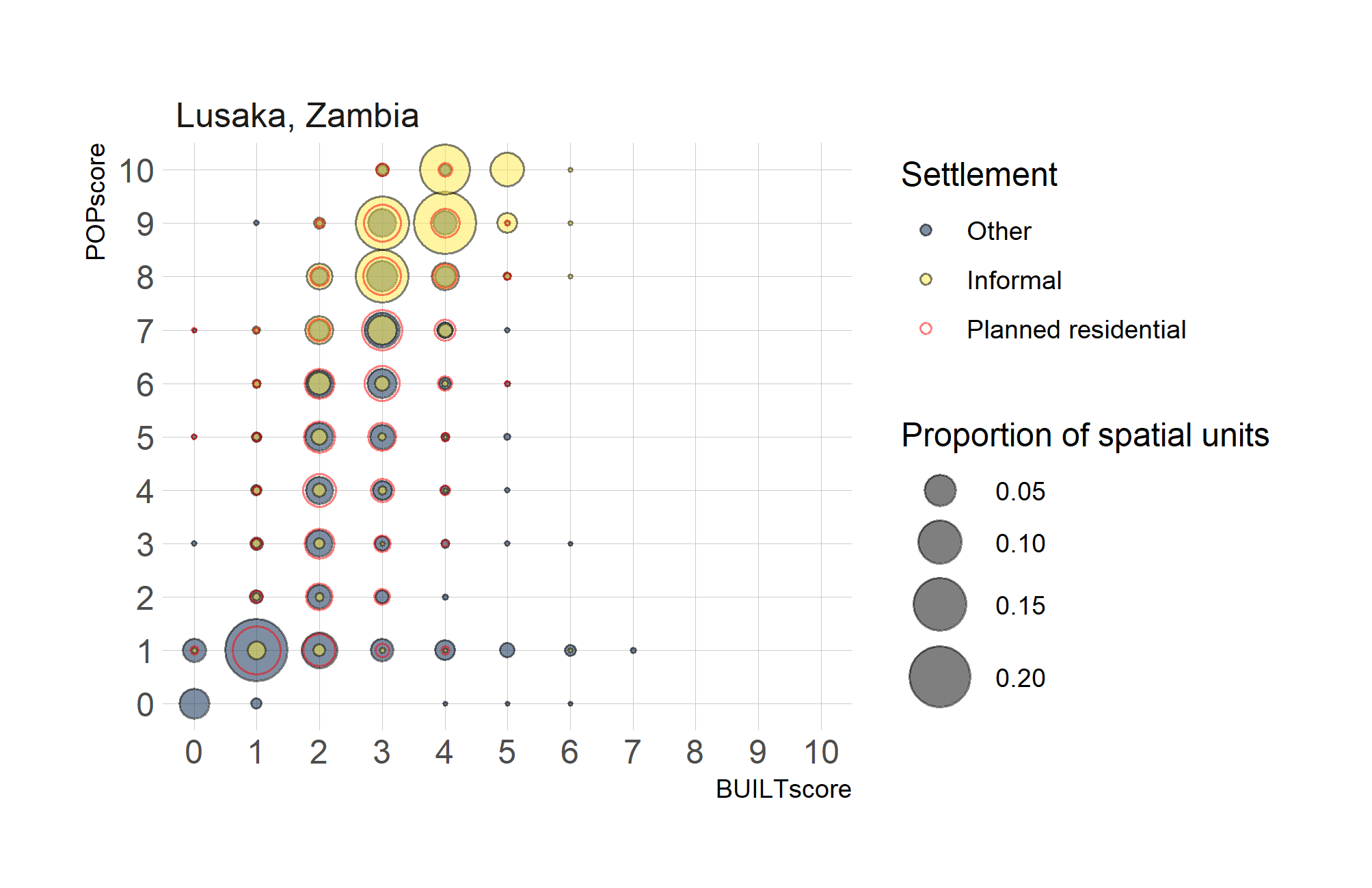

Supplement: Supplementary file 3 — Supplementary Figure 2 [file 41597_2022_1799_MOESM3_ESM.png]
